# Longitudinal choroidal thickness changes among Chinese young adults with various refractive errors

**DOI:** 10.3389/fmed.2023.1036087

**Published:** 2023-03-22

**Authors:** Fang Duan, Ziyan Chen, Zhenyu Wang, Jiayu Deng, Anna C.H. Yeo, Adeline Yang, Björn Drobe, Yee Ling Wong, Xiang Chen

**Affiliations:** ^1^State Key Laboratory of Ophthalmology, Zhongshan Ophthalmic Center, Sun Yat-sen University, Guangdong Provincial Key Laboratory of Ophthalmology and Visual Science, Guangzhou, China; ^2^Education & Professional Services, Essilor AMERA Pte Ltd, Singapore, Singapore; ^3^R&D AMERA, Essilor International, Singapore, Singapore

**Keywords:** choroid thickness, refractive error, young adult, subfoveal, parafoveal

## Abstract

**Purpose:**

To determine the characteristics of longitudinal choroidal thickness (CT) and axial length (AL) changes in a group of Chinese young adults with various refractive errors.

**Methods:**

In this 2 year prospective cohort study, a total of 291 (314 enrolled at baseline) Chinese medical freshmen aged 18 to 22 years (mean age, 18.7 ± 0.9 years) underwent eye examinations at baseline and follow-up visits, including cycloplegic refraction, ocular biometry measurements, and swept-source optical coherence tomography. Choroidal thickness measurements were performed at nine locations in the macular area.

**Results:**

At baseline, the CT and AL was significant differences among groups of emmetropia, mild to moderate myopia and high myopia. During a two-year period, there were significant differences found in the changes of the subfoveal CT (*p* < 0.001) and parafoveal CT of 7 locations between emmetropia and mild to moderate myopia, and the changes of the subfoveal CT (*p* = 0.002) and parafoveal CT of 6 locations between emmetropia and high myopia. But there were no differences for AL and SE (*p* > 0.05). The multivariable linear regression analysis showed that baseline subfoveal CT (per 1 μm) was a significant factor affecting the changes of subfoveal CT (*p* < 0.001), whereas age, gender, and baseline AL were not significantly associated.

**Conclusion:**

The longitudinal change in CT varies with refractive errors in Chinese young adults aged 18 to 22 years over a two-year period. The changes of subfoveal CT were significantly associated with the baseline subfoveal CT, but not associated with baseline AL.

## Introduction

Myopia is a leading cause of visual impairment worldwide (1–3). Myopic eyes are characterized by scleral extracellular matrix remodeling, hypoxia-inducible factor signaling promoted through myofibroblast transdifferentiation (4). The choroid may have a potential role in controlling eye growth during childhood, as it regulates the scleral extracellular matrix *via* transmission of retinal signals to the sclera (5). It is also a source of scleral growth regulators that induces changes in ocular growth in response to visual stimuli. For example, environmental factors such as light exposure can influence ocular growth through choroidal thickness (CT) changes. A study found that daily morning light therapy was associated with increased choroidal thickness in healthy young adults (6). CT, which is highly influenced by human hormonal levels and age, is an important parameter for studying myopia and myopic pathology development. Also, CT has been suggested to be a predictor of the effectiveness of orthokeratology treatment (7).

Numerous cross-sectional studies in children and adults have reported significantly thinner CT in eyes with myopia, compared with those with emmetropia and hyperopia (8–17). Also, recent studies consistently showed that thinner CT was associated with older age in adults (18–20). On the other hand, several other studies found that a thicker CT was associated with older age in healthy pediatric eyes without significant refractive error (21, 22). This suggests the likelihood of mechanisms involving choroidal changes during myopia progression in the human eye. However, the cross-sectional nature of these studies has limited the temporality of the associations. Therefore, it is crucial to determine if the choroid thickens or thins over time in children and adults under different refractive error conditions. One recent longitudinal study revealed that significant choroidal thickening over 18 months in children aged 10 to 15 years (21). In contrast, rapid thinning of the choroid was reported among newly developed myopes aged 6 to 18 years (22), while another study found choroidal thinning that occurred early during myopia progression in a group of children aged 7 to 12 years (23). The inconsistent findings indicate the need for further investigation regarding the relationship between refractive error and the change in choroidal thickness across various age groups.

There is a lack of data on the changes in choroidal thickness in young adulthood, among those aged 18 to 22 years, during the period when adolescents transition to adults, which may provide insights on the discrepancies between children and adults. Therefore, this cohort study aims to examine the changes in choroidal thickness and axial length over 2 years in a group of Chinese young adults with various refractive errors.

## Methods

### Participants and study design

This prospective cohort study followed a group of Chinese medical students over a 2-year period (from 2017 to 2019) to determine the changes in refractive errors and ocular biometry among Chinese young adults. The study participants were first-year university students from the School of Medicine, Sun Yat-sen University, Guangzhou, China. Those students with systemic diseases, intraocular lens or aphakic eyes, astigmatism ≥1.50 diopters (D) in both eyes, anisometropia ≥1.00 D, severe ocular abnormalities, dysfunction in binocular vision, history of ocular surgery, or those who underwent medical therapy (such as atropine treatment), were excluded from the study. A total of 367 first-year medical students in 2017 were screened, of which 307 (83.7%) were eligible and participated in the study at baseline. Of 307 participants, 291 (94.8%) attended the 2-year follow-up visit. Ethics approval was obtained from the human ethics committee of the Zhongshan Ophthalmic Center. The study was conducted in accordance with the Declaration of Helsinki. For each participant, written consent was obtained prior to baseline examination.

### Refraction and biometry measurements

Trained ophthalmologists and optometrists conducted the following procedures at the Zhongshan Ophthalmic Center Clinic for all visits. All the subjects underwent cycloplegic autorefraction using (LR-2, canon, Japan) on both eyes, after three instillations of 1% tropicamide eyedrops, the mean of 5 consecutive measurements was recorded as the final value for each eye. Refractive error was analyzed using spherical equivalent (SE), defined as the spherical power plus half of the cylindrical power. Emmetropia was defined as SE > −0.5 D, low to moderate myopia was defined as SE between −0.5 D to > − 6.0 D, and high myopia was defined as SE worse than or equal to −6.0 D.

Monocular visual acuity was measured at 4 m with the Logarithmic Visual Acuity Chart. Best-corrected visual acuity (BCVA) was also measured. The mean of 5 ocular biometry measurements was collected, including axial length (AL) and anterior chamber depth (ACD), using the Lenstar LS-900 (Haag-Streit AG, Koeniz, Switzerland). The mean of 3 consecutive intraocular pressure (IOP) was measured with a non-contact tonometer (TOPCON CT-80A/DKT-18).

### Optical coherence tomography

According to a previously established method, Swept-source OCT (DRI-OCT-1, Topcon, Japan) was used to evaluate CT in both eyes after cycloplegia (24). In detail, only the clear OCT images with a quality score of greater than 45 of 160 were included in the analyses. The SS-OCT used a tunable laser with a center wavelength of 1,050 nm as a light source, an 8-μm axial resolution and 10-μm transverse resolution in tissue. One trained examiner performed the OCT examinations during the hours of 10 AM to 3 PM to minimize the influence of diurnal variation. CT was defined as the vertical distance between Bruch’s membrane and the choroidal-scleral interface. A three-dimensional macular scanning was performed, the ETDRS grid was applied in the final analysis of CT, and the mean CT in each grid sector was calculated by built-in software. A built-in software completed segmentation of different layers on the OCT images and construction of topographic maps. When the process was inaccurate, leading to measurement artifacts, manual segmentation was carried out. The scan protocol was repeated three times consecutively on the same visit. The participant and device were repositioned after each scan. The diameters for the inner circle (subfoveal), middle circle and outer circle of the ETDRS grid were 0.5, 1.5, and 3 mm, respectively. Nine locations were measured (subfoveal and fovea 1.5 mm and 3.0 mm, in 4 directions, nasally, temporally, superiorly and inferiorly, including the central fovea).

### Statistical analysis

The right eye was used in the analysis (high correlation between the right and left eyes; pearson’s coefficient of 0.89).

Baseline characteristics were compared using one-way ANOVA or Chi-squared test among three refractive error groups. Linear mixed models were performed to calculate and compared the rate of ocular biometry change and choroid thickness change over 2 years, with follow-up duration, group and a group and duration interaction as fixed effects, and follow-up duration at participants level as random intercept. The follow-up duration was adjusted as categorical variable in the mixed-effect model. Multiple group comparisons adjustment was performed to preserve the family-wise error rate of 0.05 using Bonferroni correction. A multivariable linear regression was fitted to identify factors associated with the 2-year cumulative change in subfoveal choroidal thickness. Two-tailed *p* values less than 0.05 were considered statistically significant. All the statistical analyses were conducted using SAS 9.4 software (SAS Institute Inc., Cary, NC, United States).

## Results

Two hundred and twelve (72.9%) of 291 participants had mild to moderate myopia, whereas only 20 (6.9%) were emmetropic subjects and 59 (20.2%) were high myopic subjects. Of the 291 participants at baseline, 132 (45.4%) were males and 159 (54.6%) were females, with a mean age of 18.8 ± 2.4 years (Table 1). The mean age of myopia onset was 12.6 ± 2.6 years, and the age of high myopia onset was significantly younger than that of mild to moderate myopia. In addition, 47.4% of subjects had myopic parents, and 13.1% had highly myopic parents. The mean SE of the analyzed eyes was −4.0 ± 2.4 D, and the mean AL was 25.4 ± 1.3 mm. The CTs at different locations across the three refractive error groups were showed in Table 1. In general, emmetropic eyes showed the thickest CT, followed by mild to moderate myopic eye and high myopic eyes (Table 1).

**Table 1:** Baseline characteristics of Chinese medical students.

	All (*n* = 291)	Emmetropia (*n* = 20)	Mild to moderate myopia (*n* = 212)	High myopia (*n* = 59)	*p*-Value
Gender, *n* (%)					0.665
Male	132 (45.4)	11 (55.0)	95 (44.8)	26 (44.1)	
Female	159 (54.6)	9 (45.0)	117 (55.2)	33 (55.9)	
Age, years	18.8 (2.4)	19.2 (0.6)	18.8 (2.4)	18.6 (2.6)	0.643
Age of myopia onset, years*	12.7 (2.6)	NA	13.2 (2.3)	10.6 (2.4)	<0.001
Number of myopic parents (%)					0.005
0	156 (53.6)	13 (65.0)	121 (57.1)	22 (37.3)	
1	93 (32.0)	5 (25.0)	68 (32.1)	20 (33.9)	
2	42 (14.4)	2 (10.0)	23 (10.8)	17 (28.8)	
Number of highly myopic parents (%)					0.610
0	253 (86.9)	18 (90.0)	186 (87.7)	49 (83.1)	
1	34 (11.7)	2 (10.0)	24 (11.3)	8 (13.6)	
2	4 (1.4)	0 (0.0)	2 (0.9)	2 (3.4)	
Spherical equivalent, D*	−4.0 (2.4)	0.3 (0.5)	−3.4 (1.4)	−7.5 (1.4)	<0.001
Axial length, mm*	25.4 (1.3)	23.5 (0.7)	25.2 (1.0)	26.8 (0.9)	<0.001
Intraocular pressure, mmHg*	17.7 (2.5)	17.0 (2.6)	17.6 (2.5)	18.1 (2.5)	0.254
Lens thickness, mm*	3.5 (0.2)	3.6 (0.2)	3.5 (0.2)	3.5 (0.2)	0.113
K1 (horizontal), D*	42.6 (1.3)	42.7 (1.3)	42.6 (1.3)	42.5 (1.4)	0.850
K2 (vertical), D*	43.7 (1.4)	43.5 (1.4)	43.6 (1.4)	43.9 (1.4)	0.212
Choroid thickness, µm*					
Subfoveal	221.3 (68.4)	295.6 (61.3)	221.7 (66.5)	194.4 (58.3)	<0.001
Nasal, 1.5 mm	199.5 (63.1)	262.7 (64.5)	200.8 (60.5)	173.6 (56.3)	<0.001
Nasal, 3 mm	159.3 (56.0)	214.5 (62.8)	160.8 (52.8)	135.1 (50.9)	<0.001
Superior, 1.5 mm	227.0 (59.5)	277.3 (43.8)	226.6 (58.5)	211.1 (59.1)	<0.001
Superior, 3 mm	235.9 (55.9)	271.4 (40.8)	235.3 (56.0)	226.1 (55.9)	0.007
Temporal, 1.5 mm	233.6 (63.6)	300.4 (51.8)	233.4 (61.6)	211.9 (59.4)	<0.001
Temporal, 3 mm	237.5 (57.7)	290.1 (40.2)	237.3 (57.0)	220.3 (55.1)	<0.001
Inferior, 1.5 mm	221.5 (67.9)	291.1 (70.9)	221.9 (65.9)	196.6 (57.6)	<0.001
Inferior, 3 mm	217.3 (65.2)	283.5 (62.7)	217.5 (63.6)	194.3 (56.4)	<0.001

^*^ Shown is mean (standard deviation).

No statistically significant differences were found in the changes of SE, AL, keratometric power, lens thickness, IOP among emmetropic, mild to moderate myopic and high myopic groups (Table 2). However, the changes of CT over a two-year period among groups of various refractive errors exhibited significant differences (Table 2). All of them, the subfoveal choroid showed thinner over time in a two-year period. The emmetropic subjects showed the significant choroidal thinning, compared with mild to moderate myopic subjects (*p* < 0.001) and highly myopic subjects (*p* = 0.005). Similarly, significant differences were also found in the changes of parafoveal CT, the emmetropic subjects showed the significant choroidal thinning in five locations compared with mild to moderate myopic subjects and three locations compared with highly myopic subjects. However, there were no significant differences between moderate myopic subjects and highly myopic subjects.

**Table 2:** Estimated change rate of ocular biometry and choroid thickness in Chinese medical students using linear mixed model.

	Emmetropia (*n* = 20)	Mild to moderate myopia (*n* = 212)	High myopia (*n* = 59)	*P*-value † (Mild to moderate myopia vs. Emmetropia)	*P*-value^†^ (High myopia vs. Emmetropia)	*p*-value^†^ (High myopia vs. Mild to moderate myopia)
	Change rate (95% CI) *					
Ocular biometry						
Spherical equivalent, D/y	−0.11 (−0.19, −0.03)	−0.11 (−0.13, −0.08)	−0.14 (−0.18, −0.09)	>0.999	>0.999	0.902
Axial length, mm/y	0.04 (0.01, 0.07)	0.02 (0.01, 0.03)	0.04 (0.02, 0.05)	0.821	>0.999	0.276
Intraocular pressure, mmHg/y	−0.65 (−1.05, −0.26)	−0.43 (−0.55, −0.31)	−0.60 (−0.83, −0.37)	0.897	>0.999	0.602
Lens thickness, mm/y	0.01 (−0.01, 0.03)	0.02 (0.01, 0.02)	0.01 (0.003, 0.02)	>0.999	>0.999	>0.999
K1 (horizontal), D/y	−0.03 (−0.09, 0.03)	0.004 (−0.02, 0.01)	−0.04 (−0.08, −0.01)	>0.999	>0.999	0.225
K2 (vertical), D/y	0.01 (−0.07, 0.09)	−0.02 (−0.04, 0.01)	0.002 (−0.05, 0.04)	>0.999	>0.999	>0.999
Choroid thickness reduction, μm/y						
Subfoveal	−10.46 (−15.62, −5.30)	−0.51 (−2.10, 1.07)	−0.93 (−3.93, 2.07)	<0.001	0.005	>0.999
Nasal, 1.5 mm	−4.52 (−9.17, 0.12)	−0.24 (−1.67, 1.19)	−1.24 (−3.95, 1.46)	0.252	0.692	>0.999
Nasal, 3 mm	−4.26 (−7.95, −0.57)	0.17 (−0.96, 1.31)	−0.86 (−3.01, 1.29)	0.074	0.356	>0.999
Superior, 1.5 mm	−7.67 (−12.46, −2.88)	0.32 (−1.15, 1.79)	−1.09 (−3.88, 1.70)	0.006	0.060	>0.999
Superior, 3 mm	−5.49 (−9.90, −1.07)	0.86 (−0.49, 2.22)	0.63 (−1.94, 3.20)	0.022	0.057	>0.999
Temporal, 1.5 mm	−10.37 (−15.16, −5.59)	−0.45 (−1.92, 1.02)	−2.36 (−5.15, 0.43)	<0.001	0.014	0.701
Temporal, 3 mm	−6.95 (−11.73, −2.18)	−0.89 (−2.36, 0.57)	−1.06 (−3.84, 1.72)	0.053	0.110	>0.999
Inferior, 1.5 mm	−9.72 (−14.35, −5.09)	−0.74 (−2.17, 0.68)	−2.00 (−4.70, 0.69)	<0.001	0.015	>0.999
Inferior, 3 mm	−8.74 (−12.72, −4.76)	−0.10 (−1.32, 1.13)	−1.00 (−3.32, 1.32)	<0.001	0.003	>0.999

^*^ Shown are estimated rate of change and 95% confidence interval.

^†^*p* value was calculated for interaction between group and follow-up in mixed linear model and has been adjusted for multiple comparisions to control an overall type I error of 0.05 using Bonferroni Correction.

**Figure 1:**
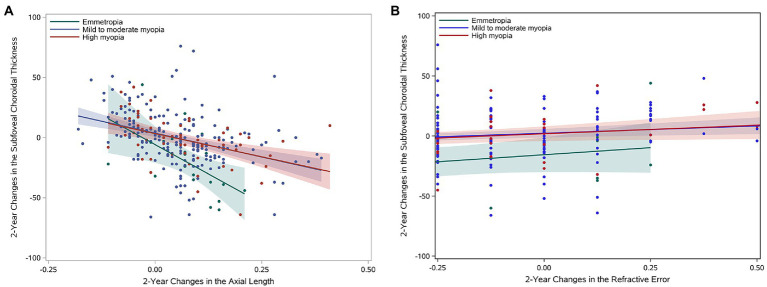
Two-year changes in the subfoveal choroidal thickness. **(A)** Linear correlation between longitudinal changes in the subfoveal choroidal thickness and changes in the axial length stratified by refractive status. **(B)** Linear correlation between longitudinal changes in the subfoveal choroidal thickness and refractive error changes.

After multivariable linear regression analysis, the baseline subfoveal CT (per 1 μm) was a significant factor in determining the changes of subfoveal CT, with a regression coefficient of −0.09 (−0.14, −0.05, *p* < 0.001), whereas age, gender, and baseline AL were not significantly associated (Table 3). In addition, negative correlations between the change in the subfoveal choroidal thickness and change in the axial length were showed in Figure 1A, Pearson correlation coefficients were − 0.56 (*p* = 0.010), −0.37 (*p* < 0.001), and − 0.44 (*p* < 0.001) in emmetropia, mild to moderate myopia and high myopia, respectively. And the positive correlations between the change in the subfoveal choroidal thickness and change in the refractive error were found for both refractive error groups (Figure 1B), Pearson correlation coefficients were 0.30 (*p* = 0.193), 0.20 (*p* = 0.005), and 0.27 (*p* = 0.042) in emmetropia, mild to moderate myopia and high myopia, respectively.

**Table 3:** Multivariable linear regression of the 2 year cumulative change in subfoveal choroidal thickness.

Parameter	Regression coefficient (95% CI)	*p* value
Age (per 1 year)	0.58 (−1.68, 0.52)	0.298
Gender		
Male	ref	
Female	−4.01 (−9.69, 1.66)	0.165
Baseline subfoveal choroidal thickness (per 1 μm)	−0.09 (−0.14, −0.05)	<0.001
Baseline Axial length (per 1 mm)	1.69 (−0.73, 4.10)	0.170

## Discussion

Our previous study of the baseline report showed that the subfoveal CT and parafoveal CT at all nine locations in myopic eyes were significantly thinner compared to emmetropic eyes (24). In the current prospective longitudinal cohort study, we found that emmetropic eyes had a faster rate of choroidal thinning than myopic eyes over a 2-year period at the subfoveal and parafoveal CT in young adults aged 18 to 22 years. The baseline subfoveal CT was a significant factor in determining the changes in subfoveal CT among young adults, while age, gender, and baseline AL were not significantly associated. This is the first study to report the longitudinal changes in CT for this young adult age group.

In our study, the change of subfoveal CT among emmetropic subjects showed significant differences compared with mild to moderate myopic subjects and highly myopic subjects. However, a study from Ulaganathan et al. reported that the subfoveal CT variations over 12 months were not significantly different between the emmetropes and myopes in 34 young adults aged 18 to 30 years (25). The difference might be explained by small sample size and short follow-up time. In addition, the baseline subfoveal CT was associated with longitudinal change in subfoveal CT among young adults, which indicated that CT still played an important role in the eye development in adulthood.

The CT was thickest at temporal and thinnest at the nasal macular region for both myopes and non-myopic at baseline. The emmetropic subjects in our study showed the greatest magnitude of parafoveal choroidal thinning at five locations compared with mild to moderate myopic subjects and three locations compared with highly myopic subjects. Our analysis of the parafoveal choroid revealed a similar magnitude of decrease in choroidal thickness over time across the parafoveal region as was observed in the subfoveal region among groups. In the presence of concurrent subfoveal choroidal thinning, the non-uniform thinning of the parafoveal choroid in young adults may be linked to the anatomy of posterior pole of the eye.

In general, changes in CT varies with age (8, 21–23), with limited literature available for the young adult group. In a study from healthy Japanese children aged 3.6 to 5.8 years, the mean central CT was significantly reduced after a 1.5-year period (26). Among children and adolescents with normal ocular growth and development, CT either thickened significantly over time (21, 22, 27, 28) or had no significant change (23). Choroidal thinning related to rapid axial eye growth with the increasing of AL and SE was found to be associated with myopia development and progression, particularly in children and adolescents with high myopia (21–23, 28, 29). Some cross-sectional studies reported that CT decreased significantly with older age in adulthood (8, 9, 18–20), with more thinning typically observed in older adult populations (> 60 years) (8, 18). Our results showed that the CT of emmetropic eyes become thinner at a faster rate than that of myopic eyes, despite no significant differences in changes in SE & AL between refractive error groups in young adulthood. This outcome indicates that the changes in CT may become independent of refractive error developments following the end of adolescence, after which the CT may only start to become much thinner during old age.

Our study did not find any significant difference in the two-year changes of AL and SE within and among groups of emmetropes, mild to moderate myopes, and high myopes. Interestingly, the emmetropic subjects in our study showed the greatest magnitude of subfoveal choroidal thinning over a 2-year period, compared with mild to moderate myopic subjects and highly myopic subjects. This suggests that self-regulatory feedback mechanisms might be involved in the regulation of a slow but significant choroidal thinning at the fovea during steady eye growth, especially in emmetropic eyes (30, 31).

The strengths of our current study included the longitudinal data analysis of subjects with a high follow-up rate (95%), allowing greater insight into the changes in choroidal thickness with age and eye growth in an age range of 18 to 22 years. The main limitations of our study include the relatively small sample size with unequal numbers of emmetropic, mild to moderate myopic and highly myopic subjects, and short follow-up time, as emmetropia is in the limited number in this group of population. The recruited participants were Chinese medical students. It may limit the generalizability of our findings to Chinese young adults in general from various regions in China and other countries.

In conclusion, it is the first time that the longitudinal change in CT varies with diverse refractive errors in Chinese young adults aged 18 to 22 years over a two-year period were reported. The CT of emmetropic eyes at both subfoveal and parafoveal locations thinned at a relatively faster rate than that of myopic eyes, with no significant differences in changes in SE and AL between refractive error groups in young adulthood. The baseline subfoveal CT was a significant factor for evaluating the longitudinal change in subfoveal CT among young adults. Choroid seems continually play a role in this age group of adults’ refractive error development.

## Data availability statement

The raw data supporting the conclusions of this article will be made available by the authors, without undue reservation.

## Ethics statement

The studies involving human participants were reviewed and approved by the human ethics committee of Zhongshan Ophthalmic Center. The patients/participants provided their written informed consent to participate in this study.

## Author contributions

FD, XC: conception and design. JD, ACY, AY, and YW: data collection. ZW: analysis and interpretation. ZC, FD, and XC: manuscript draft, review, and revision. All authors contributed to the article and approved the submitted version.

## Funding

This work was supported by funds from Natural Science Foundation of Guangdong province (2020A1515011364) and Essilor International.

## Conflict interest

ACY is an employee of Essilor AMERA, and AY, BD, and YW are employees of Essilor International.

The remaining authors declare that the research was conducted in the absence of any commercial or financial relationships that could be construed as a potential conflict of interest.
